# Case Report: No radiographic evidence of disease for 45 months in a patient with recurrent clear cell renal cell carcinoma treated with third-line combination therapy utilizing toripalimab and axitinib

**DOI:** 10.3389/fimmu.2026.1885805

**Published:** 2026-09-09

**Authors:** Ye Yang, Juan Lang, Zhongkui Xiong

**Affiliations:** 1 Department of Pathology, Shaoxing People’s Hospital, Zhejiang, Shaoxing, China; 2 School of Medicine, Shaoxing University, Zhejiang, Shaoxing, China; 3 Department of Radiation Oncology, Shaoxing Second Hospital, Zhejiang, Shaoxing, China

**Keywords:** axitinib, immunotherapy, programmed death-ligand 1, renal cell carcinoma, targeted therapy, toripalimab, vascular endothelial growth factor

## Abstract

Renal cancer is estimated to have accounted for 2.4% of all incident cancer cases worldwide in 2018. Renal cell carcinoma (RCC) is the most prevalent adult renal cancer, with the clear cell subtype accounting for 75-85% of cases and displaying pronounced genetic and biological heterogeneity. In the presence of metastatic disease, approximately 80% of patients are not expected to survive beyond one year. Immune checkpoint inhibitors targeting the programed death 1 (PD-1)/PD-L1 axis have been demonstrated to elicit durable clinical responses in patients with RCC. Subsequent therapeutic strategies for immunotherapy-naïve clear cell RCC, as recommended in the NCCN Guidelines Version 1.2026, include no preferred options; other recommended regimens encompass axitinib or lenvatinib in combination with pembrolizumab, cabozantinib with or without nivolumab, everolimus, lenvatinib, and nivolumab with or without ipilimumab. Currently, published evidence supporting the use of toripalimab in combination with axitinib as the third-line treatment for recurrent RCC remains limited. Thus, we report a case of recurrent clear cell RCC in which the patient achieved an overall survival of 14 years and sustained no radiographic evidence of disease for 45 months following third-line therapy with toripalimab in combination with axitinib. Based on the findings in this case, toripalimab combined with axitinib may be an option for relapsed clear cell RCC. Nevertheless, several limitations of this case report warrant acknowledgment: The diagnosis of recurrence was established based on imaging findings rather than confirmation via repeat biopsy and histopathological evaluation. As a case report, the level of evidence remains limited, and the reliability of the conclusions requires further validation in future studies.

## 1 Introduction

As is well documented, renal cancer was estimated to account for 2.4% of all incident cancer cases worldwide in 2018 (1). Approximately 295,000 new cases of renal cancer are diagnosed worldwide annually, with corresponding deaths exceeding 130,000 (2). Notably, renal cell carcinoma (RCC) is the most prevalent adult renal cancer (3), with the clear cell subtype accounting for 75-85% of all renal cancer cases and displaying marked genetic and biological heterogeneity (4). At present, surgical resection remains the cornerstone of management for localized clear cell RCC (4). Approximately one-third of patients with RCC who undergo nephrectomy present with metastatic disease (5). Additionally, the 5-year recurrence rate of RCC is clinically significant, ranging from 2.2% to 58.1% according to established risk factors, including tumor size, histologic subtype, and other relevant clinical characteristics (4). In the presence of metastatic disease, approximately 80% of patients are not expected to survive beyond one year (2).

Clear cell RCC is frequently characterized by robust angiogenesis, a process intimately linked to tumor growth and metastatic propensity (6). Microvessel density (MVD) was significantly higher in clear cell RCC tissues (101.64 ± 23.00) than in matched normal renal tissues (32.52 ± 7.85), as quantified at 200× magnification (7). MVD was also significantly elevated in the clear cell subtype of RCC compared with non–clear cell subtypes (811.2 versus 529.2 vessels/mm²) (8). In a test cohort comprising 209 clear cell RCC cases, the relative microvessel area (MVA) was 10.1% ± 6.3%, and the MVD was 416.8 ± 252.8 vessels/mm²; MVA and MVD showed a statistically significant positive correlation (9). A larger relative MVA was significantly associated with higher histologic grade, advanced tumor stage, presence of distant metastasis, sarcomatoid differentiation, and shorter tumor-specific survival (9). Moreover, activation of the vascular endothelial growth factor (VEGF) signaling pathway is frequently observed in RCC (10). It is worthwhile emphasizing that advanced or metastatic RCC is resistant to conventional chemotherapy (10). Consequently, VEGF receptors represent promising therapeutic targets for pharmacologic intervention (10). For instance, sorafenib and axitinib have been approved by the U.S. Food and Drug Administration for the treatment of metastatic RCC (11, 12). In the randomized phase 3 trial (NCT00678392), investigator-assessed median PFS was significantly longer with axitinib than sorafenib (8.3 versus 5.7 months; HR = 0.656), though median OS, a secondary endpoint, did not differ significantly between groups (20.1 vs. 19.2 months; HR = 0.969). These results support axitinib as a viable second-line treatment for metastatic RCC (13).

Programmed death-ligand 1 (PD-L1) expression is frequently upregulated and has been associated with poor prognosis and enhanced tumor aggressiveness in clear cell RCC (6). PD-L1 expression was observed in 24.2% of clear cell RCC subtypes compared to 10.9% of non-clear cell subtypes across six studies involving a total of 1,323 patients (14). In patients with RCC, PD-L1 expression was significantly associated with primary tumor stage, regional lymph node involvement, distant metastases, nuclear grade, and histologic tumor necrosis (15). Immune checkpoint inhibitors (ICI) targeting the programed death 1 (PD-1)/PD-L1 axis have been shown to elicit durable clinical responses in patients with RCC (16). Among patients with previously treated advanced RCC, overall survival (OS) was significantly longer with nivolumab, a PD-1 inhibitor, compared to everolimus in a randomized, open-label, phase 3 trial (17). PD-1/PD-L1 inhibitors have become the standard of care in the treatment of metastatic mRCC and have improved patient outcomes following failure of prior targeted therapies (18). Subsequent therapeutic strategies for immunotherapy-naïve clear cell RCC, as outlined in the NCCN Guidelines Version 1.2026, include no preferred options; other recommended regimens include axitinib or lenvatinib in combination with pembrolizumab, cabozantinib with or without nivolumab, everolimus, lenvatinib, and nivolumab with or without ipilimumab.

Toripalimab is a selective, recombinant, humanized monoclonal antibody that specifically targets PD-1 (19). A randomized, open-label, phase III study (RENOTORCH) evaluated toripalimab in combination with axitinib versus sunitinib as first-line therapy in patients with intermediate- or poor-risk unresectable or metastatic clear cell RCC. Interestingly, the median progression-free survival (PFS) was 18.0 months in the toripalimab-axitinib group, compared with 9.8 months in the sunitinib group. Besides, a trend toward improved OS was also observed in favor of the toripalimab plus axitinib combination (harzard ratio, HR = 0.61) (20). Nonetheless, at present, published evidence supporting the use of toripalimab in combination with axitinib as a third-line treatment for recurrent RCC remains scarce. Consequently, we herein report a case of recurrent RCC in which the patient achieved a 14-year OS, with sustained no radiographic evidence of disease for 45 months following third-line treatment with toripalimab in combination with axitinib, with follow-up extending to February 2026.

## 2 Case presentation

**Figure 1:**
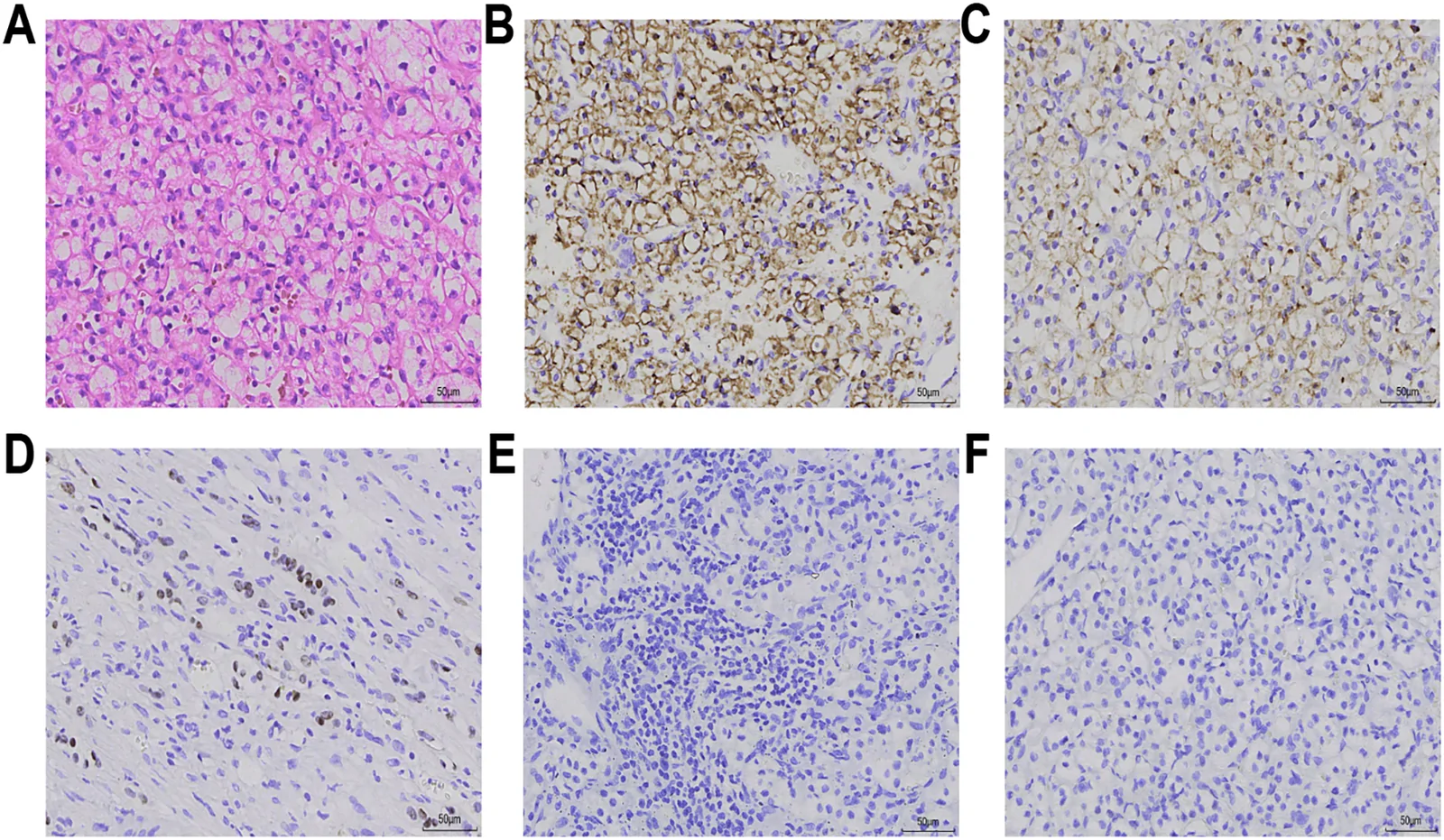
Postoperative pathological findings. Scale bar: 50 µm; magnification: ×200. **(A)** Hematoxylin–eosin (H&E) staining. The tumor was assigned a histologic grade of G2. **(B-F)** Immunohistochemical (IHC) staining results. **(B)** CA IX (membrane, +), **(C)** CD10 (membrane, +), **(D)** PAX-8 (+), **(E)** CK7 (-), **(F)** PD-L1 (-), Ventana, clone No. SP263.

A 52-year-old Chinese male was admitted on February 19, 2012, with a left renal mass and a hospital stay of four days. On physical examination, a firm, approximately 8-cm-diameter mass was palpable in the epigastric region. The patient reported no prior diagnosis of hypertension, diabetes mellitus, occupational illness, or hereditary conditions. A radical left nephrectomy was performed under general anesthesia. Pathological examination revealed clear cell RCC of the left kidney (**Figure 1A**), with a tumor size of 8 cm × 5 cm. The ureteral resection margin was negative, and adrenal tissue was identified in the specimen. At low magnification (40×), the tumor exhibited a well-demarcated interface with the adjacent normal renal parenchyma. Tumor cells were organized in nested and alveolar architectures, with the nests surrounded by an abundant network of thin-walled blood vessels. At high magnification (400×), the tumor cells appeared polygonal or round, featuring abundant clear (eosinophilic–vacuolated) cytoplasm, distinct cell membranes, medium-sized nuclei, mildly irregular nuclear contours, readily identifiable nucleoli, and no evidence of necrosis, sarcomatosis differentiation, or rhabdoid features. Lymphovascular invasion was absent. The tumor was assigned a histologic grade of G2. Immunohistochemical staining uncovered positive expression of CA IX (membrane) (**Figure 1B**), CD10 (membrane) (**Figure 1C**), PAX-8 (**Figure 1D**), EMA and CK, negative expression of CK7 (**Figure 1E**), PD-L1 (**Figure 1F**), P53, P21, and C-erbB-2, Ki-67 positivity in less than 1% of tumor cells, and moderate positivity for non-metastatic gene 23 (nm23, ++). Postoperative pathological staging, based on the American Joint Committee on Cancer (AJCC) Seventh Edition criteria, was classified as pT2N0M0, corresponding to Stage II. Subcutaneous interferon (300 U) was administered postoperatively for 6 months. On January 28, 2019, an enhanced chest CT conducted at Shaoxing Second Hospital revealed a mass in the right lower lobe, accompanied by multiple bilateral pulmonary nodules, mediastinal lymphadenopathy, and multiple soft tissue nodules in the left renal fossa and right cardiophrenic angle. Subsequent positron emission tomography/computed tomography (PET/CT) at the First Affiliated Hospital of Zhejiang University School of Medicine demonstrated post-surgical changes consistent with prior left nephrectomy for RCC, with an irregular soft tissue density in the operative bed adherent to the adjacent pancreatic tail and displaying heterogeneous FDG uptake. Given these clinical findings, local recurrence was suspected, with possible involvement of the pancreatic tail. Additionally, a soft tissue mass was observed in the anterior basal segment of the right lower lobe near the oblique fissure, with mildly increased FDG metabolism, suggestive of metastasis. Moreover, several small subpleural and parenchymal nodules were noted bilaterally, with mild or nonsignificant FDG uptake, raising suspicion for low-metabolic metastatic lesions. Enlarged lymph nodes were also observed at the right cardiophrenic angle and within the posterior mediastinum, with marginally increased FDG metabolism. Following these findings, and in accordance with the eighth edition of the AJCC Cancer Staging Manual, the clinical stage was classified as rT1N1M1, corresponding to Stage IV disease. Disease-free survival (DFS) was 83 months. Thereafter, first-line treatment was initiated with oral targeted therapy comprising sorafenib tablets at a dose of 0.4 g twice daily. A chest CT performed on April 2, 2019, showed a significant reduction in the size of bilateral pulmonary nodules compared to the previous scan on January 28, 2019, although mediastinal lymphadenopathy persisted. Soft tissue nodules remained evident in the left renal fossa and right cardiophrenic angle. On July 9, 2019, chest CT unveiled enlargement of the right lower lobe nodule compared to the April 2 scan, whereas other pulmonary and subpleural nodules remained stable. Likewise, mediastinal lymphadenopathy persisted. The PFS achieved with first-line therapy, designated PFS1, was 5 months. Given radiological evidence of disease progression and suspected sorafenib resistance, treatment was switched to axitinib at a dose of 5 mg twice daily.

**Figure 2:**
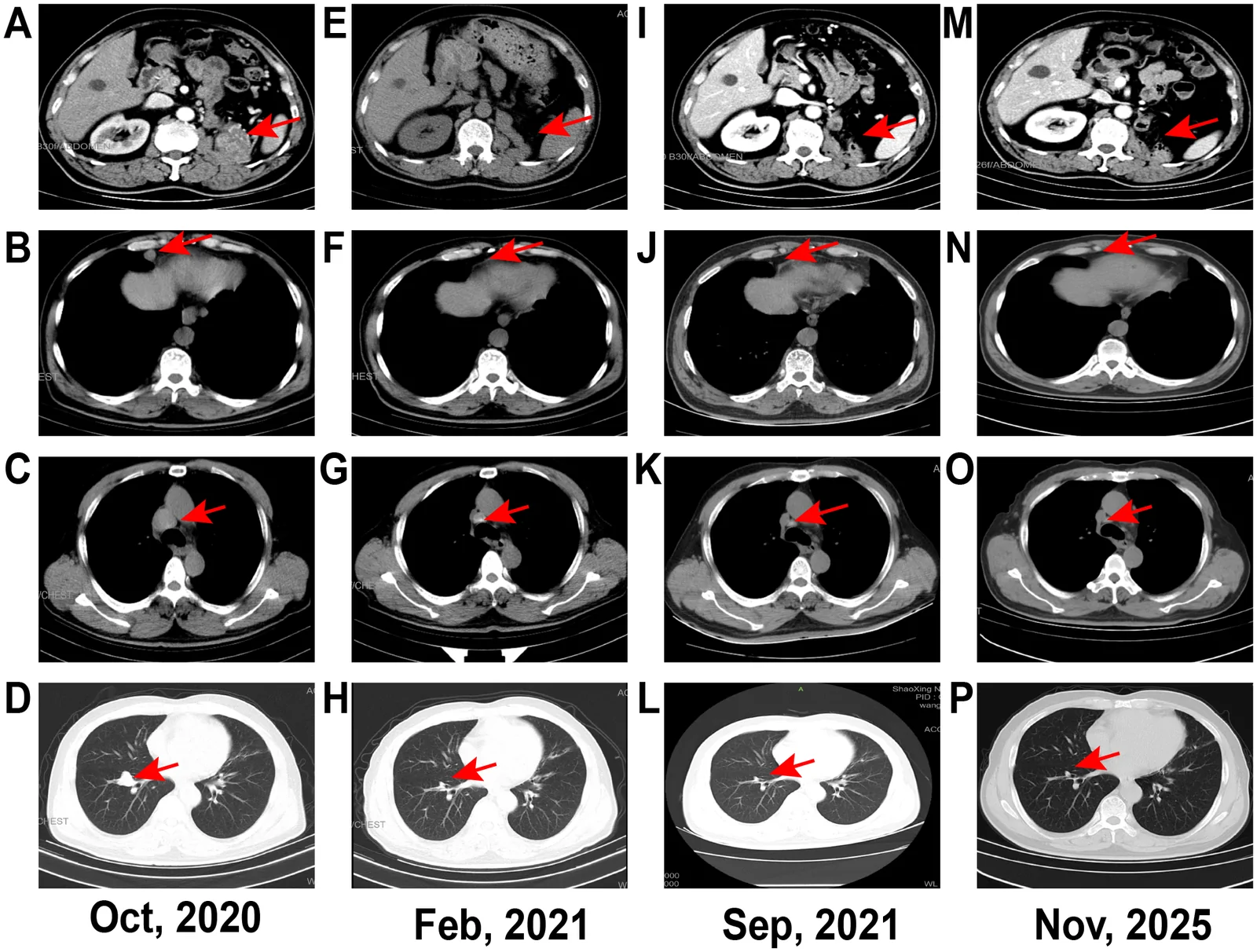
Tumor volume changes after third-line therapy assessed using CT. **(A–D)** CT scans performed in October 2020, prior to initiation of third-line therapy with toripalimab and axitinib. **(A)** An irregular, soft-tissue mass measuring approximately 65 mm × 40 mm was identified in the left renal region; enhancing nodules were also observed in the retroperitoneal regions, as indicated by the red arrow. **(B)** A nodule at the cardiophrenic angle. **(C)** Mediastinal lymphadenopathy. **(D)** Pulmonary nodules. **(E–H)** CT images acquired in February, 2021. **(E)** A substantial decrease in the volume of soft tissue mass in the left renal region. **(F)** A marked reduction of nodele in cardiophrenic angle. **(G)** Regression of mediastinal lymph nodes. **(H)** Reression of the size of pulmonary nodules located in the right upper lobe. **(I–L)** CT images performed in September, 2021. **(I)** A marked reduction in the size of the left renal lesion. **(J)** No abnormalities in cardiophrenic angle **(J)**. **(K)** No abnormalities in mediastinal lymph nodes. **(L)** No abnormalities in the right lung. **(M–P)** CT performed in November 2025 demonstrated no radiological evidence of disease.

On October 15, 2020, contrast-enhanced abdominal CT revealed an irregular, heterogeneously enhancing soft tissue mass measuring approximately 65 mm × 40 mm in the left renal region (**Figure 2A**). Besides, enhancing nodules were identified in retroperitoneal regions (**Figure 2A**). On October 16, 2020, chest CT demonstrated a nodule at the cardiophrenic angle (**Figure 2B**), mediastinal lymphadenopathy (**Figure 2C**), and pulmonary nodules (**Figure 2D**). A needle biopsy was recommended to confirm the diagnosis; however, the patient declined the procedure. Consequently, no histopathological evidence of recurrence was available. At this time point, treatment response was assessed as radiographic PD, and the progression-free survival achieved with second-line therapy, namely PFS2, was 15 months. Subsequently, combination therapy consisting of toripalimab (240 mg administered intravenously on Day 1 every 3 weeks) and axitinib (5 mg orally twice daily) was administered for 1 year, from October 2020 to September 2021. On February 3, 2021, CT images delineated a substantial decrease in the volume of soft tissue mass in the left renal region (**Figure 2E**), a marked reduction of nodule in cardiophrenic angle (**Figure 2F**), and regression of mediastinal lymph nodes (**Figure 2G**) and the size of pulmonary nodules located in the right upper lobe (**Figure 2H**). Assessment of treatment response was conducted via CT after three months of third-line therapy, revealing a radiographic partial response (PR) per the Response Evaluation Criteria in Solid Tumors (RECIST) version 1.1. On September 2, 2021, contrast-enhanced CT demonstrated a marked reduction in the size of the left renal lesion (**Figure 2I**), with the retroperitoneal lesion (**Figure 2I**) remaining minimal and stable (**Figure 2**). On September 3, 2021, chest CT showed no abnormalities in cardiophrenic angle (**Figure 2J**), mediastinal lymph nodes (**Figure 2K**), or the right lung (**Figure 2L**). On February 19, 2022, contrast-enhanced abdominal CT examination revealed postoperative changes in the left kidney, with no evidence of soft tissue lesions in this region; conversely, several small retroperitoneal lymph nodes (<1 cm short-axis diameter) were identified; none showed malignant features—e.g., central necrosis, heterogeneous or rim enhancement, or ill-defined margins. A radiographic complete response (CR) was achieved on February 19, 2022. On September 17, 2022, contrast-enhanced abdominal CT imaging revealed no evidence of soft tissue lesions within the left renal surgical bed postoperatively. Treatment-related adverse events (TRAEs) were observed, including grade 1 hepatotoxicity, nephrotoxicity and thrombocytopenia, as defined by the Common Terminology Criteria for Adverse Events (CTCAE) version 5.0 (**Supplementary Figures 1A–E**). During third-line therapy, tumor biomarkers, including carbohydrate antigen 125 (CA125), carbohydrate antigen 242 (CA242), and squamous cell carcinoma antigen (SCC), fell within their respective reference range (**Supplementary Figures 1F–H**).

**Figure 3:**
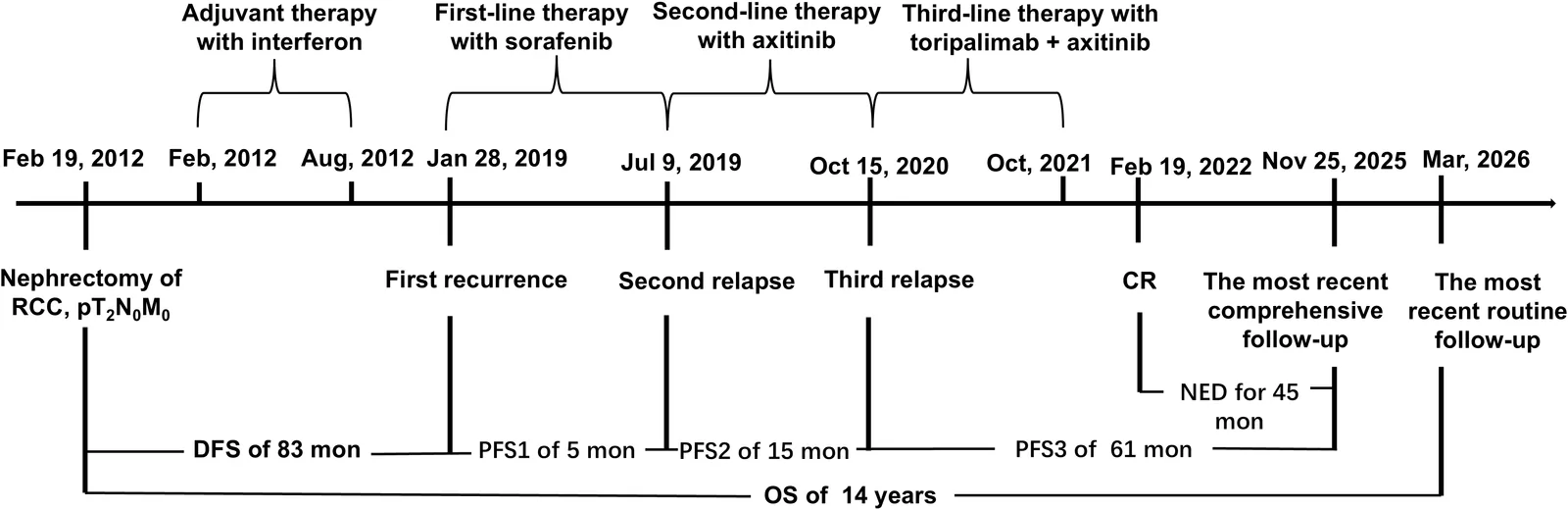
The course timeline. A recurrent clear cell RCC treated with third-line therapy comprising toripalimab and axitinib achieved a sustained state of no radiographic evidence of disease for 45 months, spanning from the radiographic CR achieved following third-line therapy on February 19, 2022, to the most recent comprehensive follow-up conducted on November 25, 2025, with a PFS3 of 61 months, spanning from the third radiographic recurrence on October 15, 2020, to the most recent comprehensive follow-up on November 25, 2025, and an OS of 14 years, calculated from nephrectomy performed on February 19, 2012, to the most recent routine follow-up visit in March 2026.

Following one year of third-line treatment with toripalimab in combination with axitinib, the patient achieved a radiographic CR. Although continued maintenance therapy, either with the dual-agent regimen or with monotherapy, may reduce the risk of disease recurrence, the patient opted to discontinue treatment and transition to surveillance-only follow-up, citing financial constraints as the primary reason. Regular and close clinical follow-up was maintained. The most recent comprehensive follow-up assessment was conducted on November 25, 2025 (**Figures 2M–P**), and the latest routine follow-up visit occurred in March 2026. Importantly, a sustained state of no radiographic evidence of disease was maintained for 45 months, spanning from the radiographic CR achieved following third-line therapy on February 19, 2022, to the most recent comprehensive follow-up conducted on November 25, 2025, with a PFS3 of 61 months, spanning from the third radiographic recurrence on October 15, 2020, to the most recent comprehensive follow-up on November 25, 2025, and an OS of 14 years, calculated from nephrectomy performed on February 19, 2012, to the most recent routine follow-up visit in March 2026, as depicted in **Figure 3**.

## 3 Discussion

In the present case, toripalimab in combination with axitinib was administered as third-line systemic therapy, with no prior exposure to immunotherapy. Anti-PD-1 monoclonal antibodies represent key ICIs for immunotherapy. In recent years, the combination of ICIs and anti-angiogenic targeted agents has emerged as a standard first-line therapeutic strategy for advanced RCC. The European Association of Urology (EAU) Guidelines Panel on RCC recommends, for first-line treatment of advanced RCC across all International Metastatic RCC Database Consortium (IMDC) risk groups, the following combinations of tyrosine kinase inhibitors (TKIs) with ICIs: axitinib plus pembrolizumab, cabozantinib plus nivolumab, and lenvatinib plus pembrolizumab. Additionally, dual immunotherapy with ipilimumab plus nivolumab is recommended for patients categorized in the IMDC intermediate- and poor-risk categories (21). In a network meta-analysis, pembrolizumab in combination with lenvatinib demonstrated rapid disease control, whereas ipilimumab plus nivolumab conferred the greatest long-term survival benefit among patients with intermediate- or poor-risk disease according to the IMDC criteria (22).

A single-arm, phase II trial recruiting patients with locally advanced clear cell RCC treated with neoadjuvant toripalimab combined with axitinib demonstrated a primary endpoint objective response rate (ORR) of 45%. In addition, tumor shrinkage was observed in 95% of patients prior to nephrectomy (23). An earlier phase 2 study (NEOTAX) involving 25 patients with clear cell RCC and inferior vena cava tumor thrombus who received neoadjuvant toripalimab plus axitinib reported that 44.0% of patients achieved a reduction in thrombus level, with no patient experiencing an increase in Mayo classification level. The median change in tumor thrombus length was -2.3 cm. The median PFS was 25.3 months, with a 1-year PFS rate of 89.1% (24). As a first-line therapy for patients with previously untreated intermediate- or poor-risk advanced RCC in the United States, toripalimab in combination with axitinib was not considered cost-effective compared with sunitinib monotherapy from the perspective of a U.S. third-party payer (25). In a retrospective propensity-matched cohort study involving patients with metastatic clear cell RCC, those receiving first-line targeted therapy demonstrated superior OS compared to patients receiving first-line immunotherapy, with median OS of 33 months and 15 months, respectively. The median PFS was 3.5 months in patients receiving a first-line combination of TKI and anti-PD-1 therapy and 14.5 months in patients who received TKI combined with anti-PD-1 following initial TKI therapy (26). According to another study, toripalimab in combination with axitinib demonstrated significantly superior efficacy compared to other immunotherapy-based combination regimens with respect to both PFS and OS (27).

A retrospective study involving 50 patients with advanced RCC 90% of whom had received at least one prior line of systemic therapy, evaluated the combination of axitinib and toripalimab. With a median follow-up duration of 11.9 months, the median PFS was 13.1 months, and the median OS had not yet been reached. The one-year OS rate was 84.6% (28). A retrospective study recruiting 57 patients with metastatic RCC who experienced disease progression following VEGFR tyrosine kinase inhibitor therapy showed that second-line treatment with axitinib plus toripalimab achieved an objective response rate of 31.6%, with a median PFS of 11.7 months; the median OS had not been reached (29). A duration of ≥ 6 months of first-line VEGFR TKI treatment has been associated with improved OS in patients with metastatic RCC receiving nivolumab as second-line therapy (30). Anti-PD-1 antibody therapy administered following TKI therapy significantly restored T-cell function and enhanced cytotoxic activity against tumor cells in a syngeneic co-culture system comprising peripheral blood mononuclear cells and tumor cells (31).

Furthermore, a real-world study demonstrated that axitinib exhibits antitumor activity when administered as a third-line or later treatment in RCC (32). A retrospective cohort study conducted in England demonstrated that OS was significantly longer among patients with advanced RCCwho received cabozantinib as second-line or later therapy (≥2L), compared with those who received axitinib, following VEGFR-targeted therapy (33). As demonstrated in an open-label, randomized phase 3 trial, dovitinib exhibited antitumorigenic activity comparable to that of sorafenib in patients with RCC who had progressed following prior treatment with VEGF-targeted therapies and mTOR inhibitors (34). Third-line therapy for patients with advanced RCC currently incorporates immune checkpoint blockade agents (35).

Currently, established predictive biomarkers for ICI therapy include PD-L1 expression, mismatch repair deficiency (dMMR) or high microsatellite instability (MSI-H), pathogenic POLE mutations, tumor mutational burden (TMB), and tumor-infiltrating lymphocytes (TILs) (36–38). In the CheckMate 214 trial, PD-L1–positive patients derived the greatest clinical benefit from nivolumab plus ipilimumab versus sunitinib, with significantly improved PFS (HR = 0.48) and OS (HR = 0.45). In PD-L1–negative patients, OS was prolonged (HR = 0.73), but PFS showed no statistically significant improvement (39, 40). In a study evaluating nivolumab as second-line and later-line (i.e., third-line or beyond) therapy for metastatic RCC, consistent efficacy and tolerability were observed across later lines of treatment (41). In a real-world study (POST-NIVO) enrolling 208 patients with RCC, nivolumab monotherapy was administered as second-line treatment in 36.5% of patients, third-line treatment in 30.8%, and fourth-line or later treatment in 31.7%. The 36-month overall OS rate was 54.3%, with rates of 57.4% in the second-line group, 52.6% in the third-line group, and 52.9% in the fourth-line or later group (42). PD-L1 expression may serve as a predictive biomarker for PFS in clinical trials involving patients with metastatic RCC treated with ICIs, either as monotherapy or in combination with other therapeutic agents; however, its prognostic value for OS remains less well established, as indicated by a meta-analysis of randomized clinical trials (43). In another meta-analysis of five studies comprising a total of 4,063 patients with RCC treated with ICIs in first-line settings, OS was significantly improved in patients with PD-L1–positive tumors (44).

The scientific rationale underpinning these combination strategies arises from the dynamic crosstalk between the immune and angiogenic systems (45). Resistance of clear cell RCC to sunitinib, a VEGFR-TKI, is associated with remodeling of the tumor immune microenvironment, specifically through upregulation of PD-L1 expression on tumor-infiltrating immune cells (46). *In vitro* study demonstrated that VEGFR-TKI–resistant renal cancer cells exhibited upregulated expression of PD-L1 and mTOR signaling pathway, and displayed enhanced tumorigenic aggressiveness (47). Mechanistically, VEGF inhibitors enhance the antitumor efficacy of ICIs by attenuating tumor-driven immunosuppressive cell populations and promoting T-cell infiltration into the tumor microenvironment (48). Furthermore, PD-1 inhibitors contribute to vascular normalization by improving vascular function and inducing immune reprogramming (49). Preclinical evidence from the murine RenCa RCC model suggests that favorable control of advanced RCC progression can be achieved either through concurrent administration of VEGFR-TKIs and ICIs or through sequential treatment with ICIs followed by VEGFR-TKIs (50).

In this case, a durable 45-month state of no radiographic evidence of disease was achieved following third-line therapy with toripalimab in combination with axitinib, as confirmed at the most recent comprehensive follow-up. Contributors to this exceptional clinical outcome are outlined below. (I) The primary factors associated with long-term survival included nephrectomy, postoperative adjuvant therapy, and systemic targeted therapy, either as monotherapy or in combination with immunotherapy following disease recurrence. (II) Immunohistochemical analysis of postoperative tissue specimens revealed no detectable PD-L1 expression. The patient subsequently received adjuvant interferon-γ therapy. PD-L1 expression is regulated by multiple signaling pathways, notably the interferon-γ/signal transducer and activator of transcription 1 axis (51, 52). Adjuvant interferon-γ therapy may confer benefit for third-line immunotherapy with toripalimab. (III) High adherence to scheduled follow-up visits constituted a critical contributor to prolonged patient survival. An adverse prognostic factor was the pathological subtype. Clear cell RCC patients demonstrated inferior OS, with a 5-year OS rate of 62.7%, compared to 72.6% in patients with non-clear cell RCC in a population-based cohort study (53). Nevertheless, several limitations of this case report warrant acknowledgment: (I) The diagnosis of recurrence was established based on imaging findings rather than confirmation via repeat biopsy and histopathological evaluation. (II) Minimal residual disease, circulating tumor DNA, and circulating tumor cells were not employed to assess the efficacy of antitumor therapy. (III) PD-L1 expression was assessed exclusively in postoperative resection specimens and was not evaluated in biopsy specimens obtained at the time of the third disease recurrence, prior to initiation of toripalimab therapy.

In conclusion, the combination of toripalimab and axitinib may serve as a promising third-line treatment option for patients with clear-cell RCC who have not previously received immunotherapy. However, as this evidence stems from a single case report, the level of supporting evidence remains limited, and the validity of these findings warrants further confirmation in larger, prospective clinical studies.

## Data Availability

The raw data supporting the conclusions of this article will be made available by the authors, without undue reservation.

## Supplementary material

Supplementary Figure 1 Dynamic changes in laboratory test parameters. January 28, 2019, the date of the first radiologic diagnosis of recurrence, was designated as the baseline (Day 0). The red dashed line indicates the reference interval for the laboratory test results. Laboratory test parameters associated with treatment-related adverse events **(A–E)** and plasma-based tumor biomarkers **(F–H)**. Elevated serum alanine aminotransferase (ALT) **(A)** and aspartate aminotransferase (AST) **(B)** levels, consistent with CTCAE version 5.0 Grade 1 hepatotoxicity, were observed during first-line treatment. Serum creatinine **(C)** and urea **(D)** levels increased to Grade 1, as defined by the CTCAE version 5.0, during second-line therapy. Grade 1 thrombocytopenia **(E)**, as defined by the CTCAE version 5.0, was observed during third-line therapy. During third-line therapy, tumor biomarkers, including carbohydrate antigen 125 (CA125), carbohydrate antigen 242 (CA242), and squamous cell carcinoma antigen (SCC), fell within their respective reference range **(F–H)**.
