# Supplementary material for: Case Report: No radiographic evidence of disease for 45 months in a patient with recurrent clear cell renal cell carcinoma treated with third-line combination therapy utilizing toripalimab and axitinib
Source: Front Immunol. 2026 Sep 9;17:1885805. doi: 10.3389/fimmu.2026.1885805 (PMC13597443; doi:10.3389/fimmu.2026.1885805)
Supplement: Supplementary file 1 [file Table1.docx]

Table S1. Changes in target lesions during third-line therapy (mm)

|  | October 2020 | February 2021 | September 2021 | February 2022 | November 2025 |
| --- | --- | --- | --- | --- | --- |
| The longest diameter of main renal-fossa mass | 65 | 28.2 | 20.0 | - | - |
| The shortest diameter of cardiophrenic lesion | 10 | - | - | - | - |
| The shortest diameter of mediastinal lymph node | 31 | 11.6 | - | - | - |
| The longest diameter of lung nodule | 22 | 7.7 | - | - | - |
| Radiographic assessment |  | PR | PR | CR | CR |

A dash (–) denotes the absence of the finding on imaging.
